# Supplementary material for: A deep learning-based hybrid model of global terrestrial evaporation
Source: Nat Commun. 2022 Apr 8;13:1912. doi: 10.1038/s41467-022-29543-7 (PMC8993934; doi:10.1038/s41467-022-29543-7)
Supplement: Supplementary file 1 — Supplementary Information [file 41467_2022_29543_MOESM1_ESM.pdf]

Supplementary Information:
A Deep Learning-Based Hybrid Model of Global Terrestrial Evaporation

Akash Koppa[1,*], Dominik Rains[1], Petra Hulsman[1], Rafael Poyatos[2,3], and Diego G. Miralles[1]

[1]Hydro-Climate Extremes Lab (H-CEL), Ghent University, Ghent, Belgium

[2]CREAF, E08193 Bellaterra (Cerdanyola del Vallès), Catalonia, Spain

[3]Universitat Autònoma de Barcelona, E08193 Bellaterra, (Cerdanyola del Vallès), Catalonia, Spain

1 Additional Validation Figures

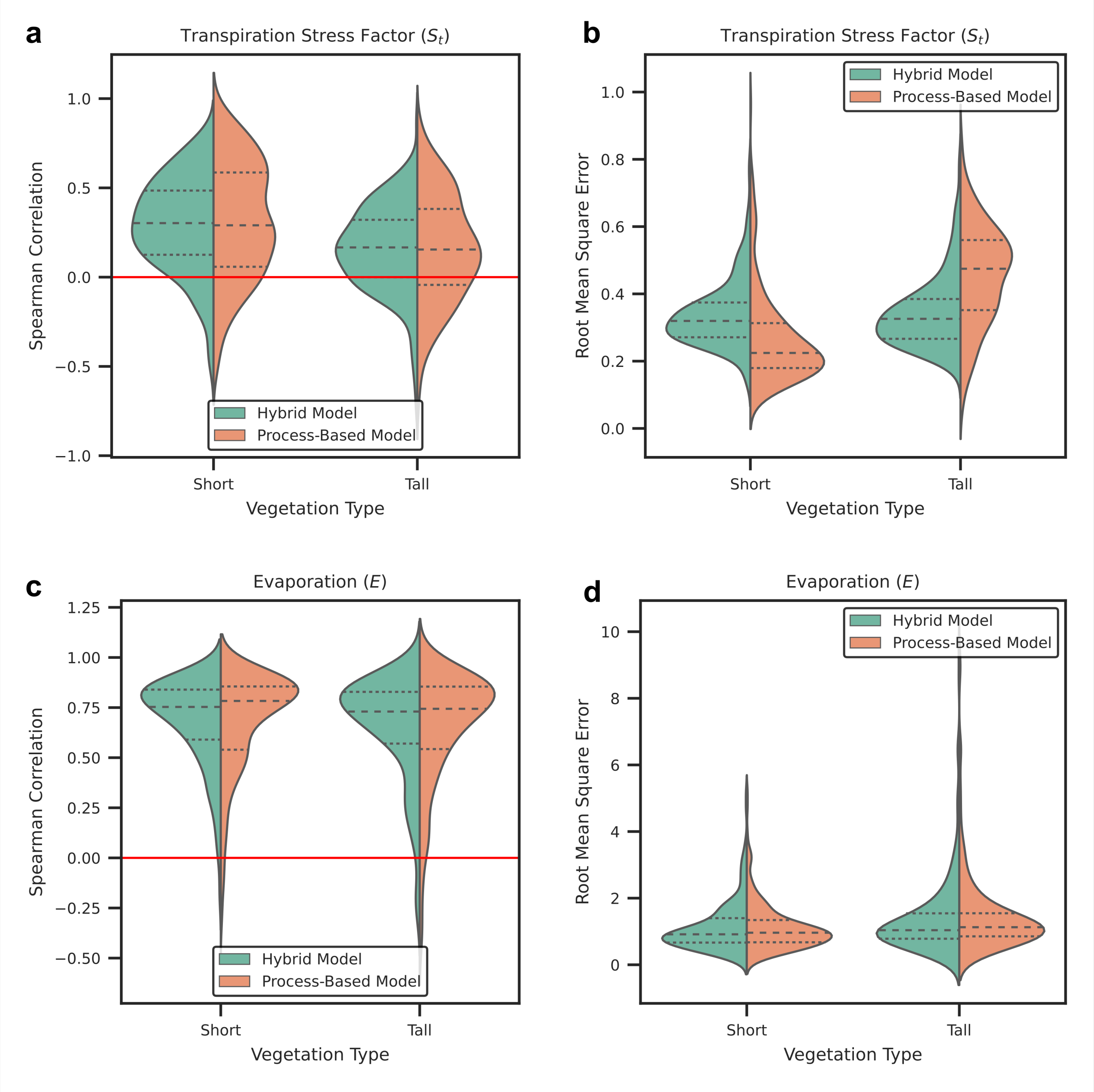

Supplementary Figure 1: Violin plots of Spearman correlation and root mean square error (RMSE) for transpiration stress factor (S_t) (**a) and b)** and evaporation (E) (**c) and d)**) calculated for the hybrid and process-based models based on observations from 368 flux towers and 90 sap flow measurement sites. For the sap flow sites, transpiration estimates (S_t) instead of E is used.

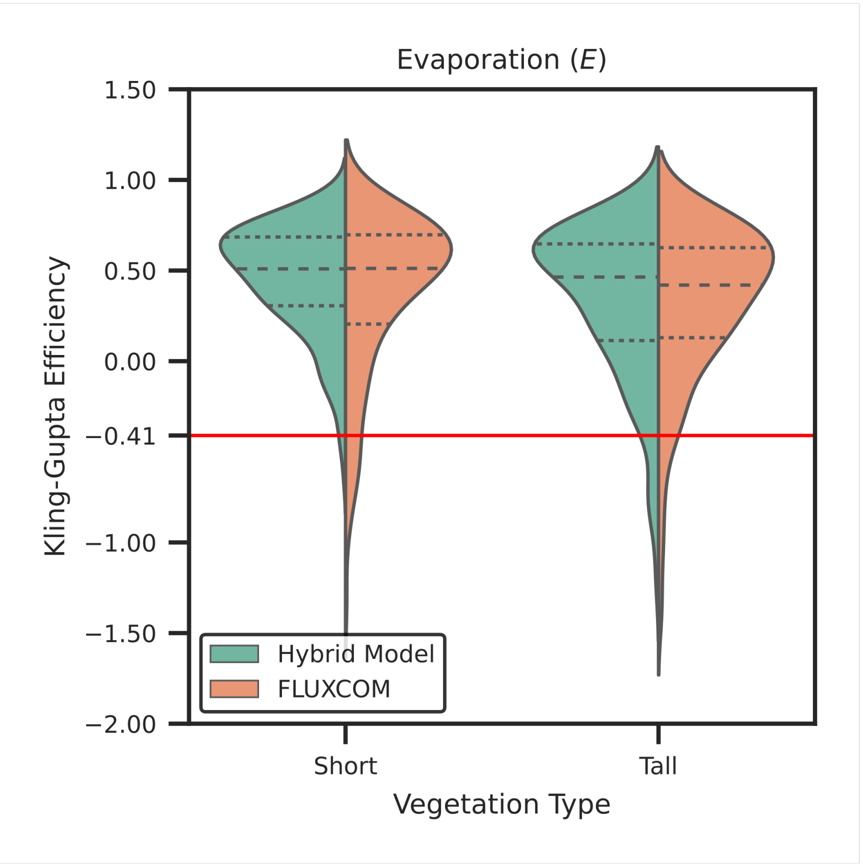

Supplementary Figure 2: Violin plot of Kling-Gupta Efficiency (KGE) for evaporation (*E*) calculated for the hybrid model and the FLUXCOM machine learning-based *E* dataset at the 368 flux towers and 90 sap flow measurement sites. For the sap flow sites, transpiration estimates (E_t) instead of E is used.

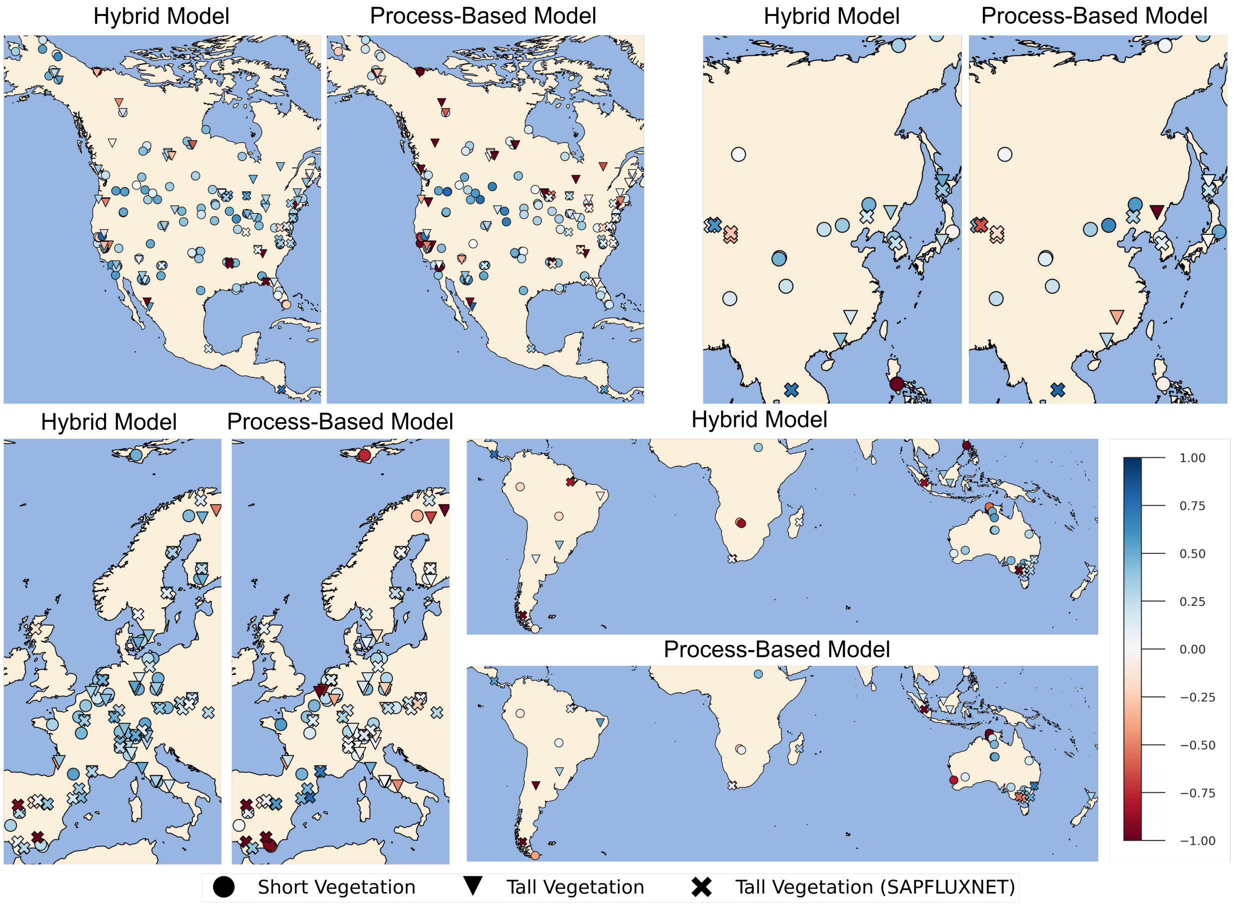

Supplementary Figure 3: Maps showing the Kling-Gupta Efficiency (KGE) metric of transpiration stress factor (S_t) calculated for the hybrid and process-based models using observations at flux tower and sap flow measurement sites in different zones (defined according to Figure 3 in the main text): North America (NA), Asia (AS), Europe (EU), Rest of the World (RW). For the sap flow sites, transpiration estimates (S_t) instead of E is used.

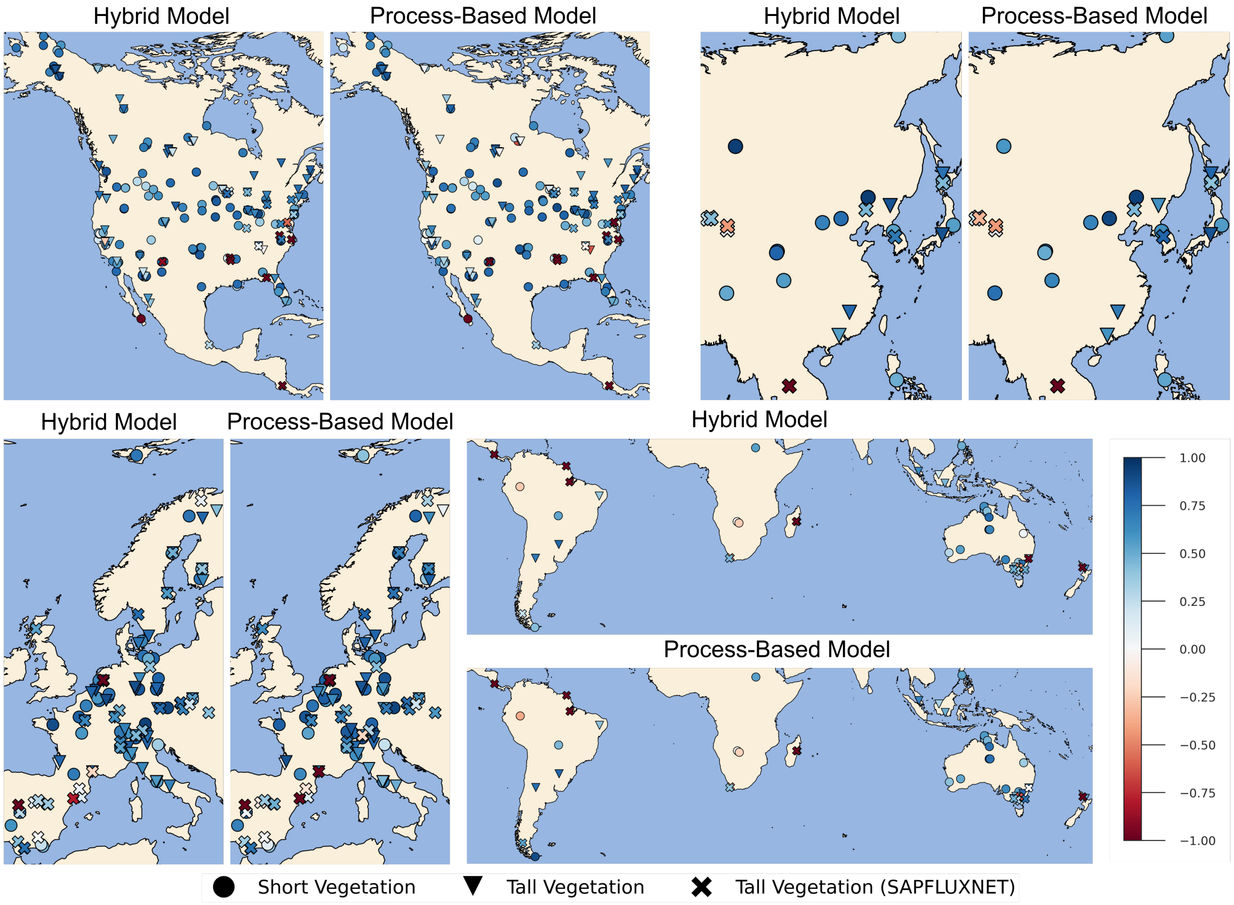

Supplementary Figure 4: Maps showing the Kling-Gupta Efficiency (KGE) metric of evaporation (E) calculated for the hybrid and process-based models using observations at flux tower and sap flow measurement sites in different zones (defined according to Figure 3 in the main text): North America (NA), Asia (AS), Europe (EU), Rest of the World (RW). For the sap flow sites, transpiration estimates (S_t) instead of E is used.

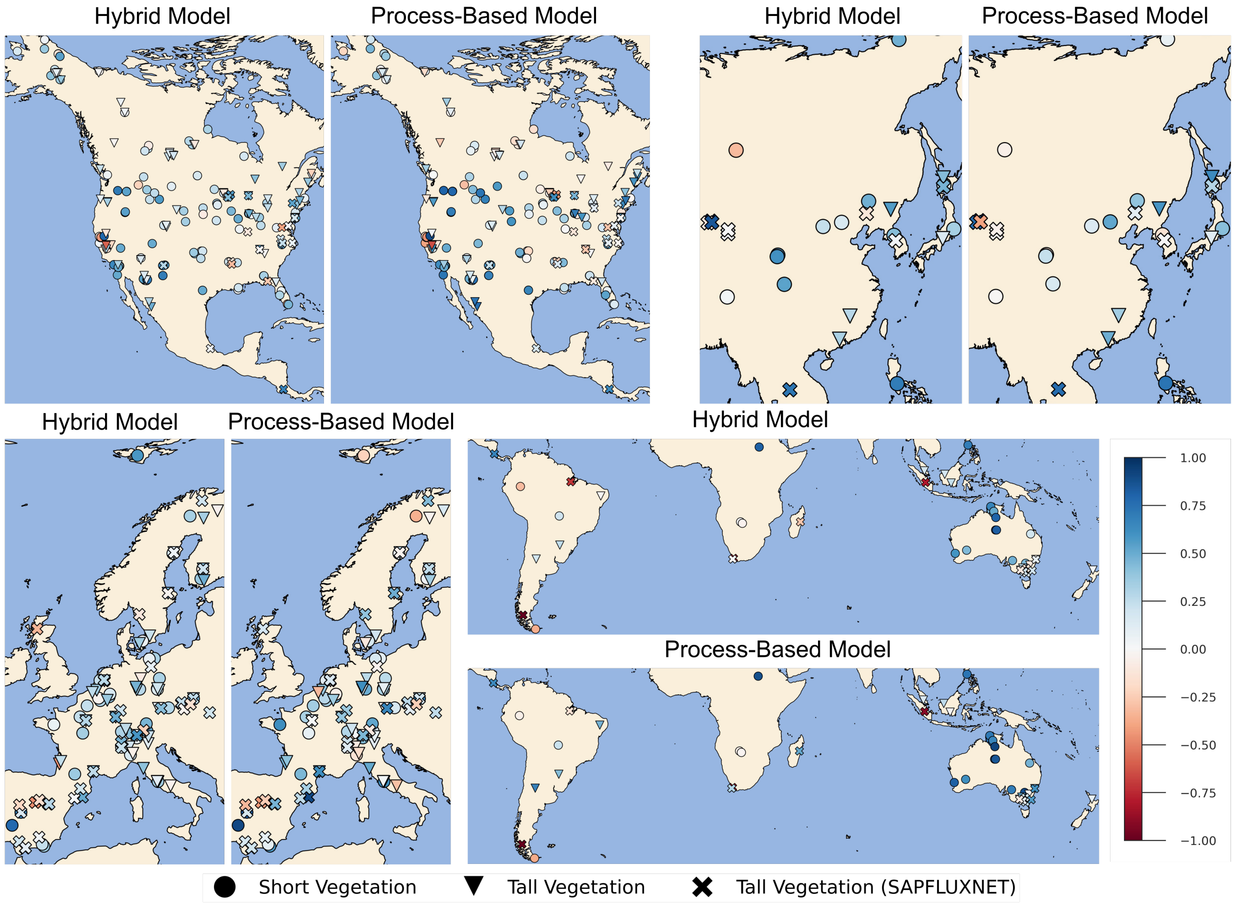

Supplementary Figure 5: Maps showing the Spearman correlation of transpiration stress factor (S_t) calculated for the hybrid and process-based models using observations at flux tower and sap flow measurement sites in different zones (defined according to Figure 3 in the main text): North America (NA), Asia (AS), Europe (EU), Rest of the World (RW). For the sap flow sites, transpiration estimates (S_t) instead of E is used.

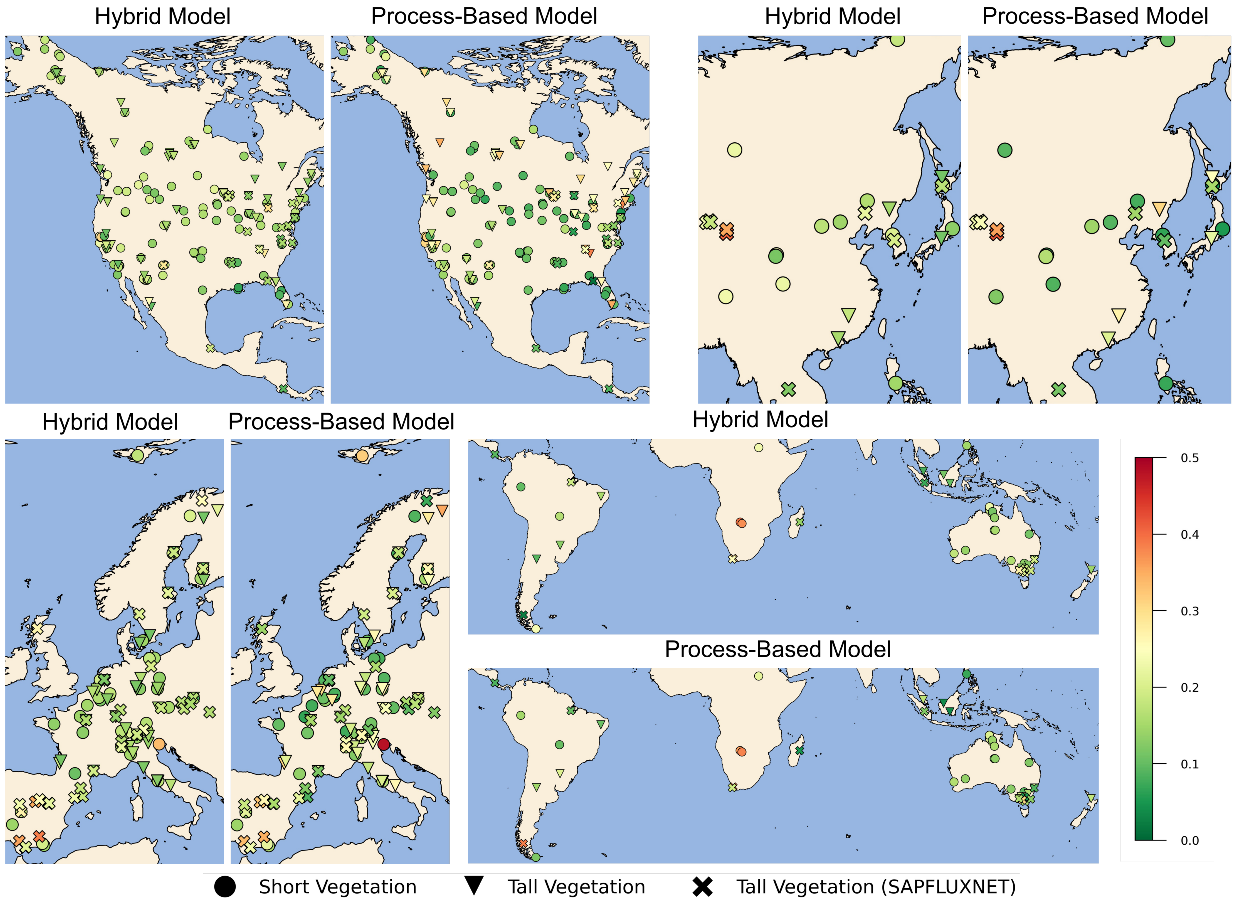

Supplementary Figure 6: Maps showing the root mean square error of transpiration stress factor (S_t) calculated for the hybrid and process-based models using observations at flux tower and sap flow measurement sites in different zones (defined according to Figure 3 in the main text): North America (NA), Asia (AS), Europe (EU), Rest of the World (RW). For the sap flow sites, transpiration estimates (S_t) instead of E is used.

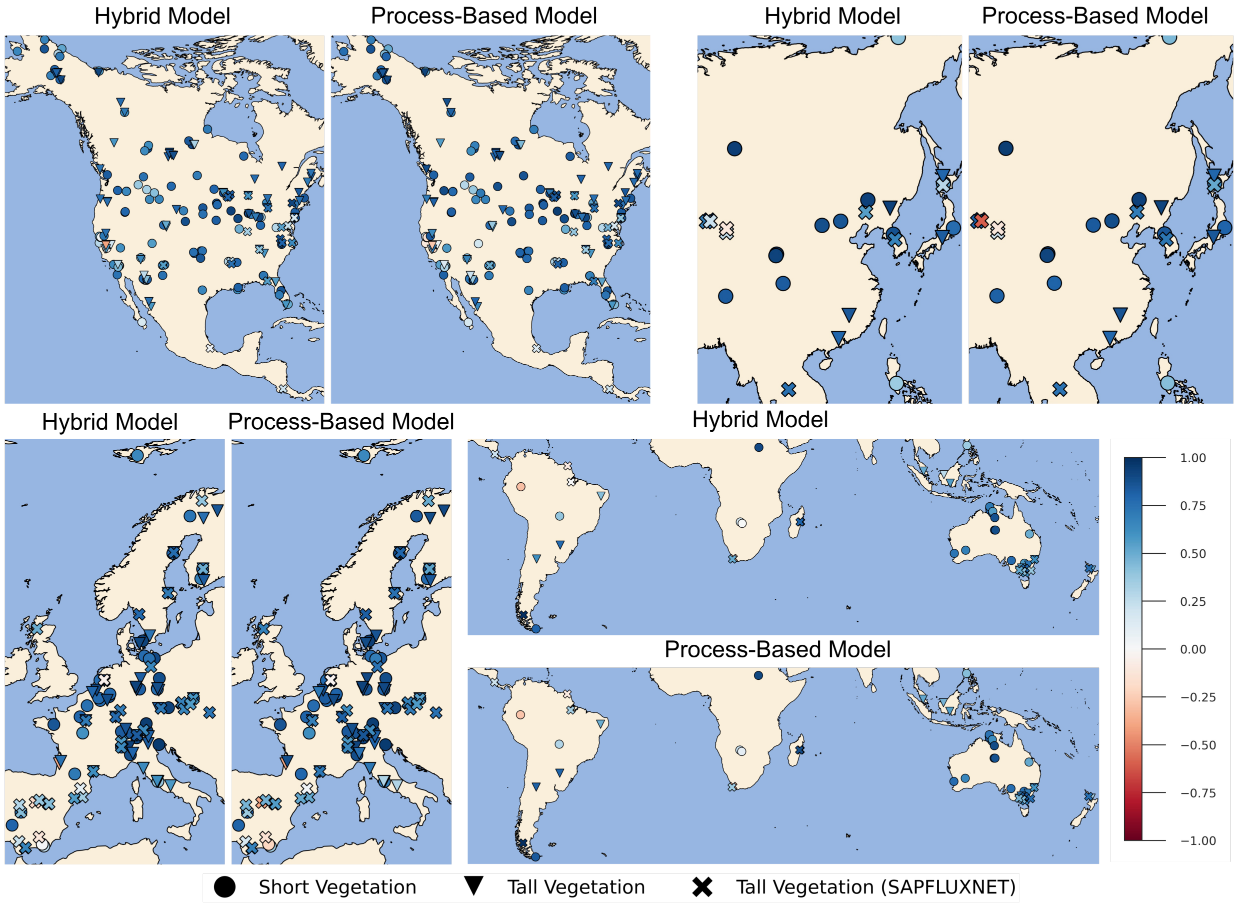

Supplementary Figure 7: Maps showing the Spearman correlation of evaporation (E) calculated for the hybrid and process-based models using observations at flux tower and sap flow measurement sites in different zones (defined according to Figure 3 in the main text): North America (NA), Asia (AS), Europe (EU), Rest of the World (RW). For the sap flow sites, transpiration estimates (S_t) instead of E is used.

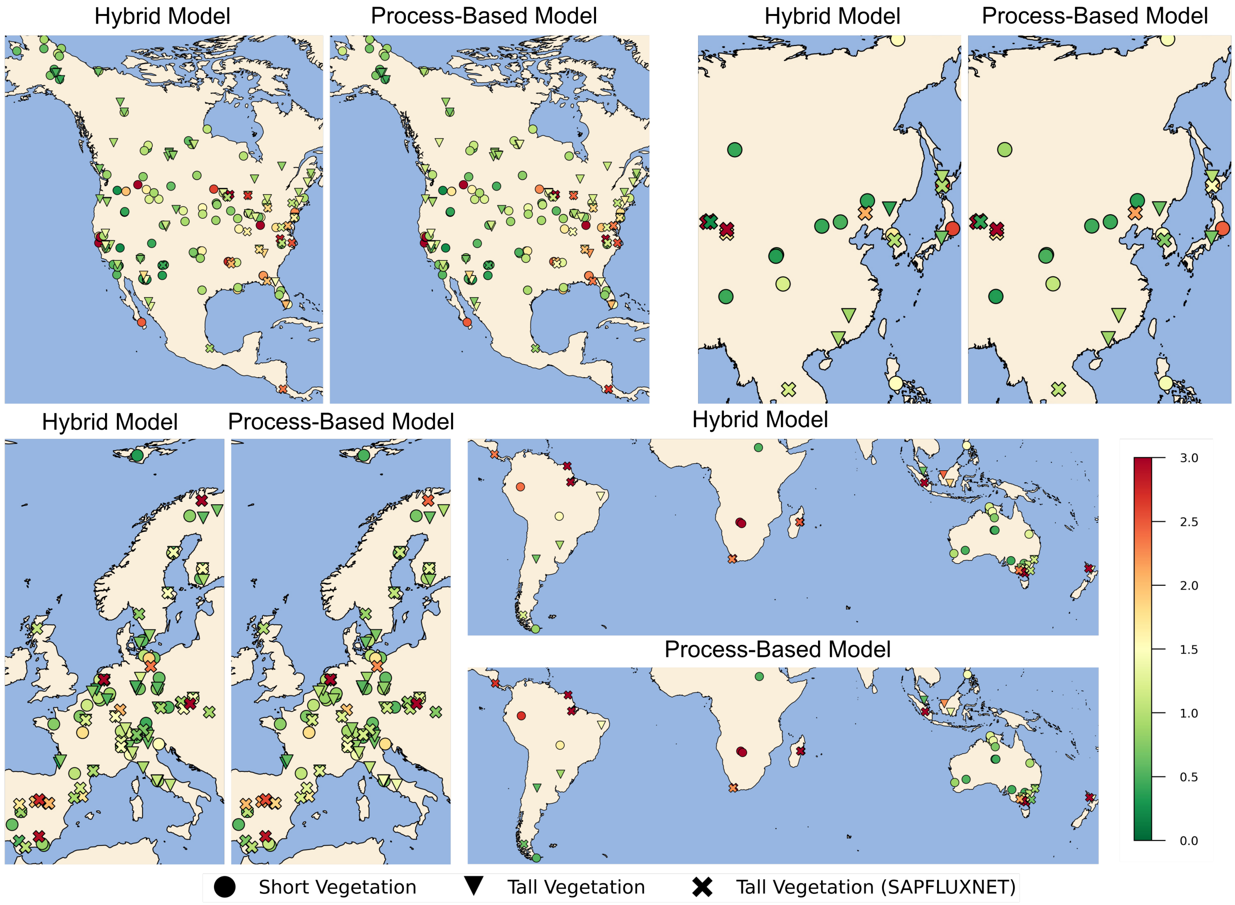

Supplementary Figure 8: Maps showing the root mean square error of evaporation (E) in mm/day calculated for the hybrid and process-based models using observations at flux tower and sap flow measurement sites in different zones (defined according to Figure 3 in the main text): North America (NA), Asia (AS), Europe (EU), Rest of the World (RW). For the sap flow sites, transpiration estimates (S_t) instead of E is used.

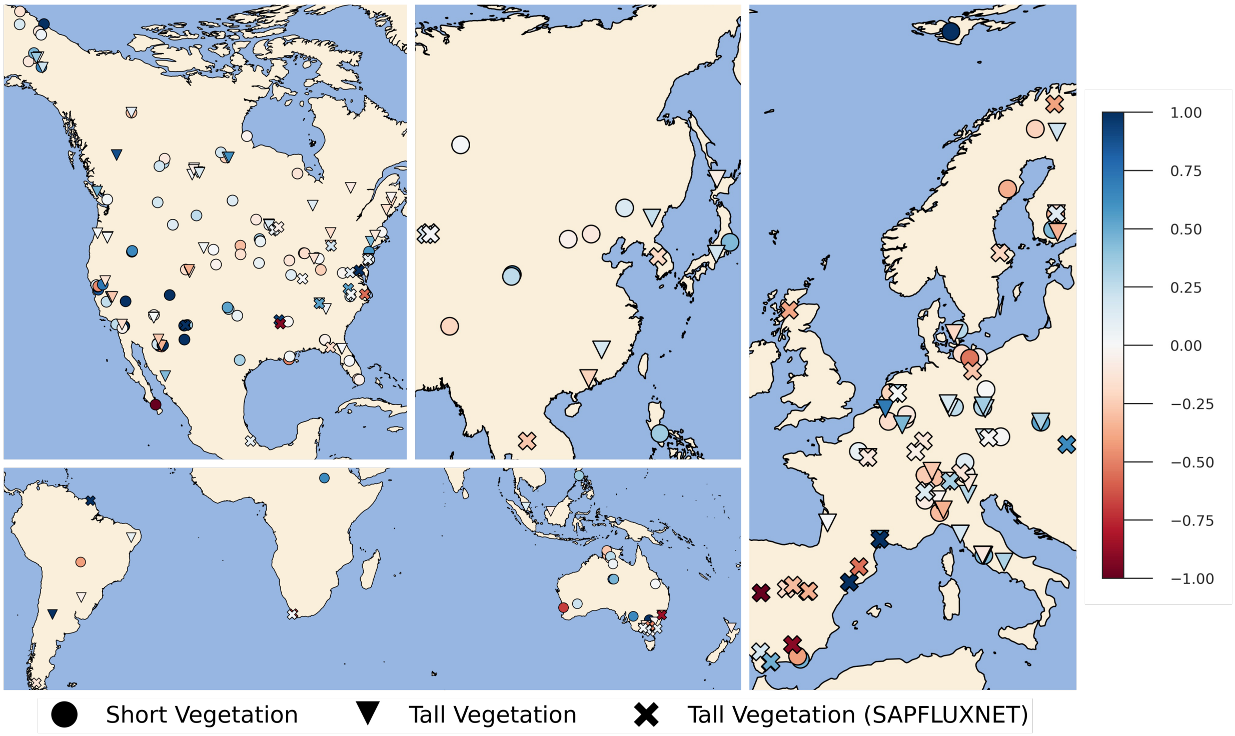

Supplementary Figure 9: Maps showing the difference in Kling-Gupta Efficiency (KGE) between the hybrid model and the FLUXCOM machine-learning based evaporation (E) dataset calculated at flux tower and sap flow measurement sites in different zones (defined according to Figure 3 in the main text): North America (NA), Asia (AS), Europe (EU), Rest of the World (RW). For the sap flow sites, transpiration estimates (S_t) instead of E is used.

2 Separation of Transpiration and Bare-soil Evaporation

The ratio of transpiration (E_t) to total evaporation (E) is empirically related to leaf area index (LAI) as

$$\frac{E_t}{E} = \alpha * LAI^{\beta} \tag{S1}$$

where α and β are regression coefficients[1]. The regression coefficients vary according to land cover class (Supplementary Table 1). We use Equation S1 with the appropriate regression parameters (Supplementary Table 1) to estimate transpiration at each of the 368 stations used for training the deep learning models.

Supplementary Table 1: Regression co-efficients used for separating transpiration and evaporation at flux tower sites

**Land Cover**	α	β
Broadleaf Forests	0.64	0.15
Needleleaf Forests	0.48	0.32
Mixed Forests	0.52	0.26
Shrub and Grasslands	0.69	0.28
Crops	0.66	0.18
Wetlands	0.65	0.21

3 Process-based Model Input Data

Supplementary Table 2: Input data for the process-based model (GLEAM). The additional input data required for the hybrid model is detailed in the Methods section of the main text

Variable	Source
Radiation	CERES L3 SYN1dg Ed4.1[2]
Air Temperature	AIRS L3 RetStd v7.0[3]
Precipitation	MSWEP v2.8[4]
Snow Water Equivalent	GLOBSNOW L3v2[5]
Vegetation Optical Depth	VODCA[6]
Surface Soil Moisture (assimilated)	ESA-CCI v5.3[7]
Vegetation Fractions	MOD44B v6.0

4 Details of the Deep Learning Model Architecture

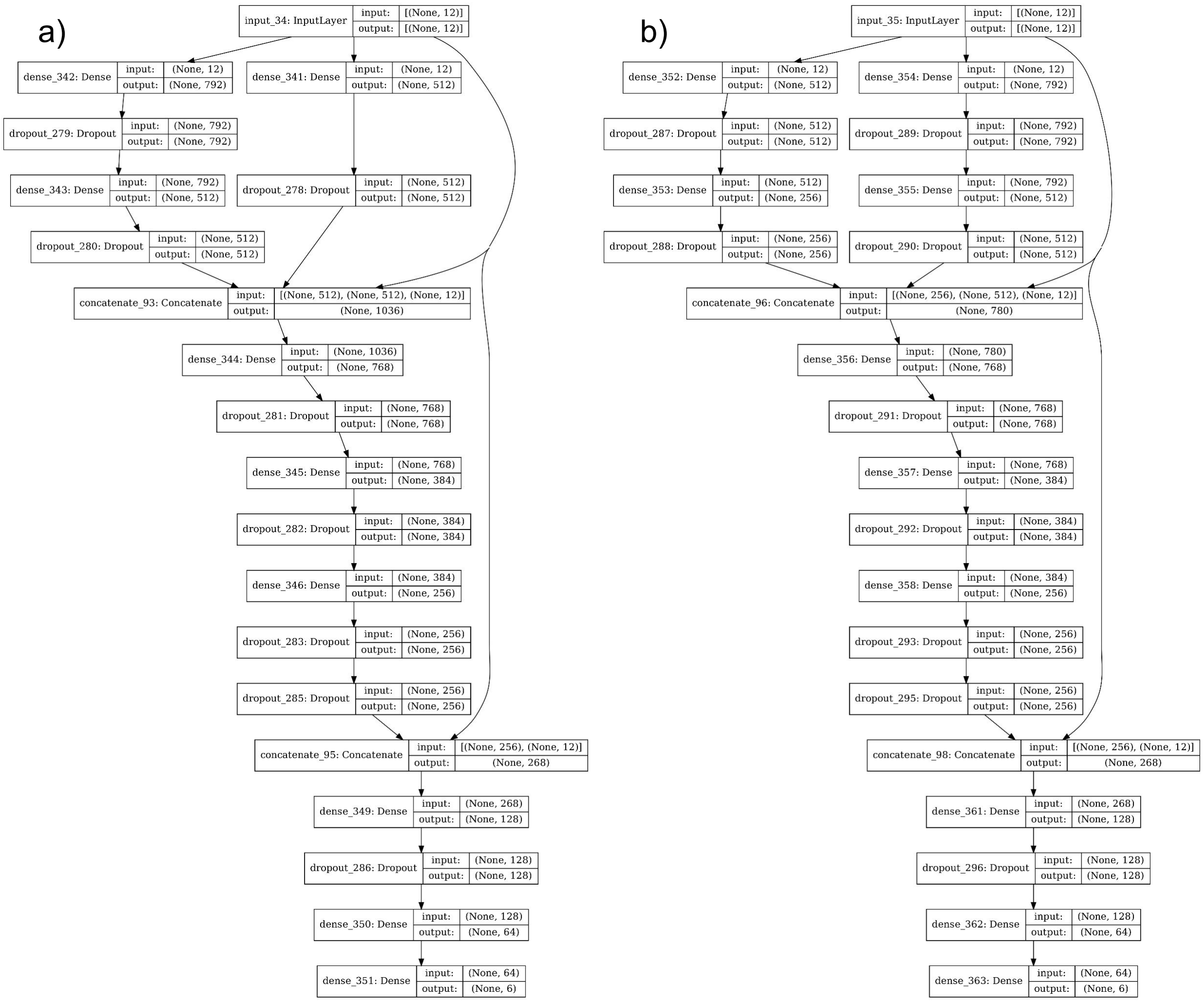

Supplementary Figure 10: Deep learning architecture for **a)** tall and **b)** short vegetation. Each block represents one layer. Here, Input layer takes in the covariates and feeds the subsequent neural network layers. The Dense layer consists of connected neurons (the number of neurons in each layer is given alongside ’output’ inside each block) and a nonlinear activation function. The activation functions used in this study are swish[8] and Gaussian error linear units (gelu)[9] (activation functions are not shown in the figure). Dropout layer is used to implement an approach to prevent overfitting which reduces the number of neurons in the input Dense layer by a random amount during training.

a

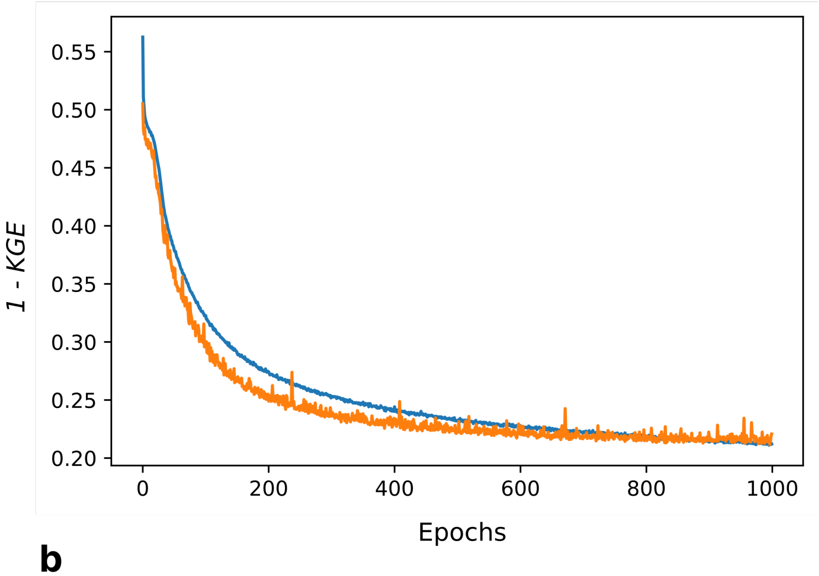

b

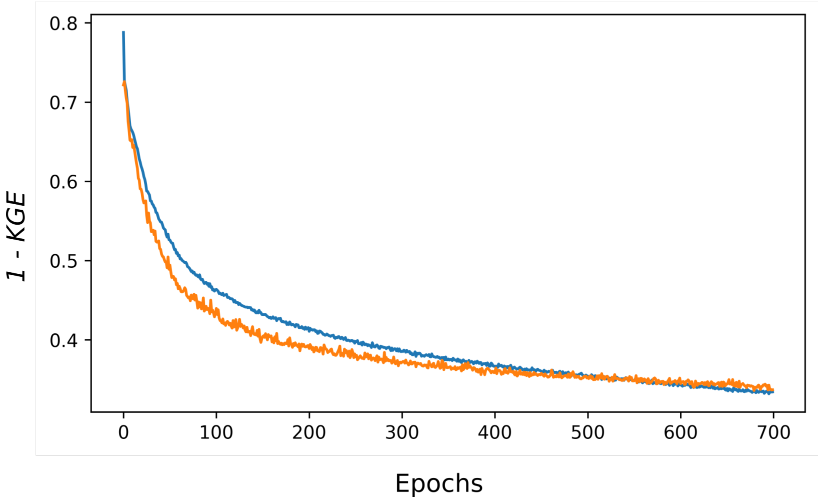

Supplementary Figure 11: Evolution of the loss function (minimization of 1 - Kling-Gupta Efficiency (KGE)) during the training of the deep learning model for **a)** short and **b)** tall vegetation. Note: The blue line represents the objective function ($1 - KGE$) for the training dataset (85% of the total data) and the orange line represents the objective function change for the validation dataset (15% of the total data). The intersection point seen in both the graphs represent the stage where the model transitions from an underfitting model to an overfitting model.

5 In-situ Measurements of Evaporation from flux towers

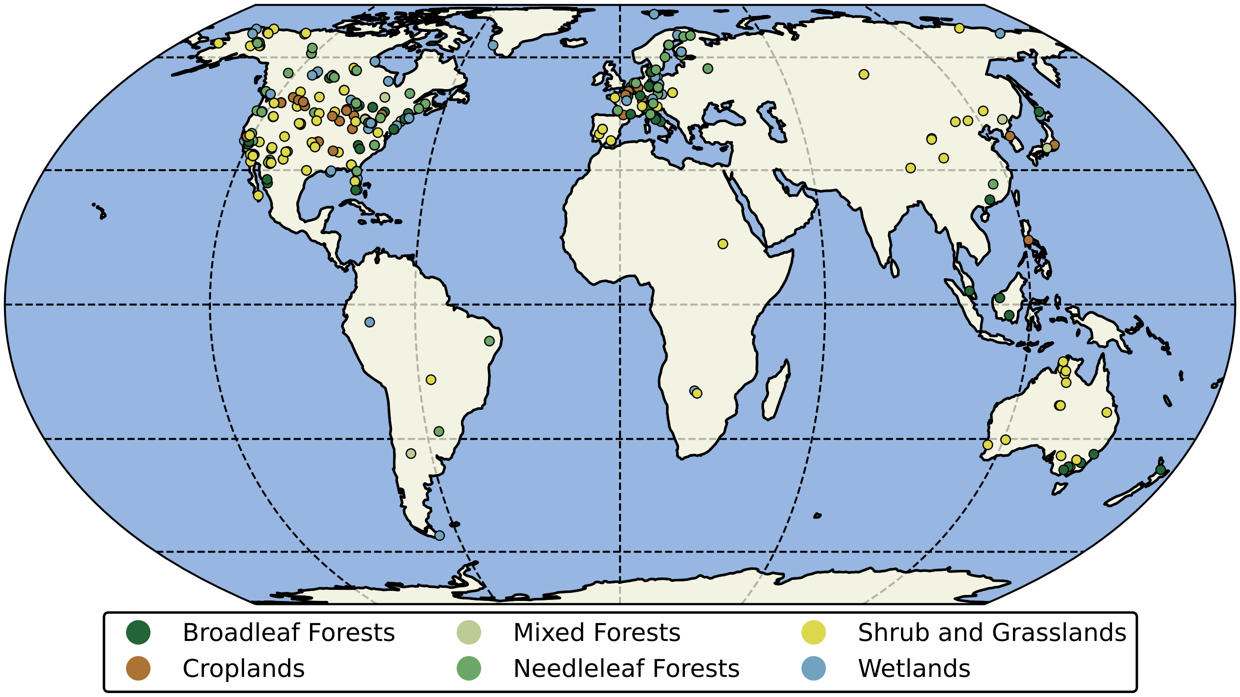

Supplementary Figure 12: Spatial distribution of the 368 flux towers used in the study. The flux towers are classified according to land cover.

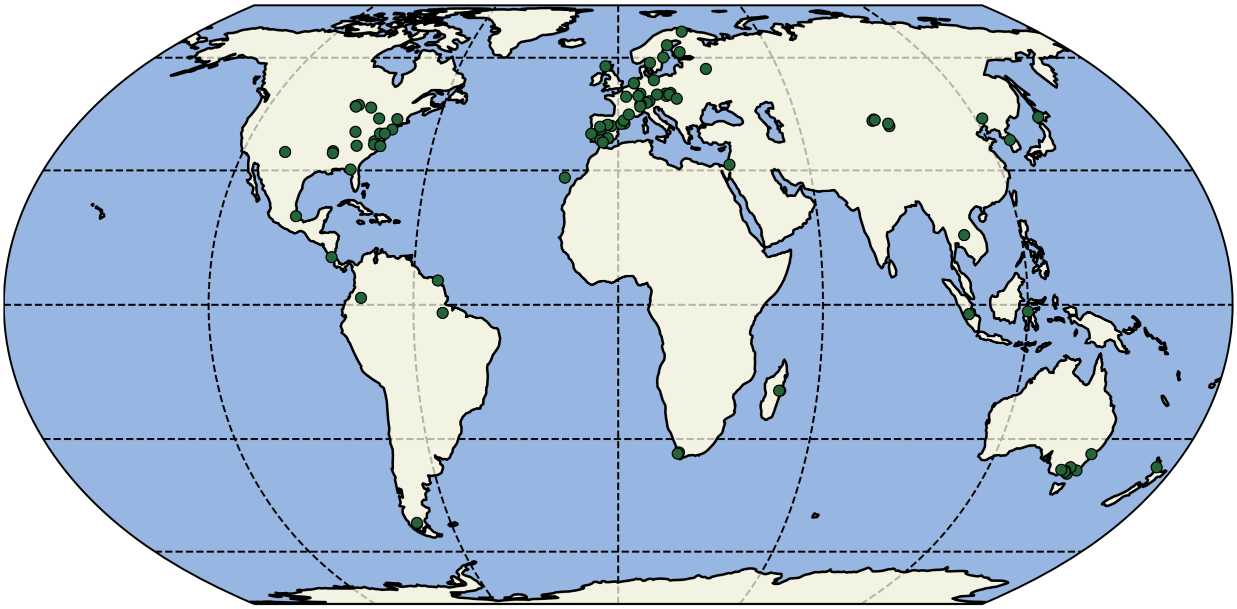

Supplementary Figure 13: Spatial distribution of the 90 sap flow measurement stations (from the SAPFLUXNET database) used in the study.

Supplementary Table 3: Meta data of the 368 stations used in this study for training and validation of the deep learning models of transpiration stress factor (S_t for tall and short vegetation).

Site ID	Latitude	Longitude	IGBP Class	Wei Class	GLEAM Class
AR-SLu	-33.4648	-66.4598	MF	Mixed Forests	Tall
AR-TF1	-54.9733	-66.7335	WET	Wetlands	Short
AR-Vir	-28.2395	-56.1886	ENF	Needleleaf Forests	Tall
AT-Neu	47.1167	11.3175	GRA	Shrub and Grasslands	Short
AU-ASM	-22.2830	133.2490	SAV	Shrub and Grasslands	Short
AU-Ade	-13.0769	131.1178	WSA	Shrub and Grasslands	Short
AU-Cpr	-34.0021	140.5891	SAV	Shrub and Grasslands	Short
AU-Cum	-33.6152	150.7236	EBF	Broadleaf Forests	Tall

AU-DaP	-14.0633	131.3181	GRA	Shrub and Grasslands	Short
AU-DaS	-14.1593	131.3881	SAV	Shrub and Grasslands	Short
AU-Dry	-15.2588	132.3706	SAV	Shrub and Grasslands	Short
AU-Emr	-23.8587	148.4746	GRA	Shrub and Grasslands	Short
AU-GWW	-30.1913	120.6541	SAV	Shrub and Grasslands	Short
AU-Gin	-31.3764	115.7138	WSA	Shrub and Grasslands	Short
AU-How	-12.4943	131.1523	WSA	Shrub and Grasslands	Short
AU-RDF	-14.5636	132.4776	WSA	Shrub and Grasslands	Short
AU-Rig	-36.6499	145.5759	GRA	Shrub and Grasslands	Short
AU-Stp	-17.1507	133.3502	GRA	Shrub and Grasslands	Short
AU-TTE	-22.2870	133.6400	GRA	Shrub and Grasslands	Short
AU-Tum	-35.6566	148.1517	EBF	Broadleaf Forests	Tall
AU-Wac	-37.4259	145.1878	EBF	Broadleaf Forests	Tall
AU-Whr	-36.6732	145.0294	EBF	Broadleaf Forests	Tall
AU-Wom	-37.4222	144.0944	EBF	Broadleaf Forests	Tall
AU-Ync	-34.9893	146.2907	GRA	Shrub and Grasslands	Short
BE-Bra	51.3076	4.5198	MF	Mixed Forests	Tall
BE-Dor	50.3119	4.9680	GRA	Shrub and Grasslands	Short
BE-Lcr	51.1122	3.8504	DBF	Broadleaf Forests	Tall
BE-Lon	50.5516	4.7462	CRO	Croplands	Short
BE-Vie	50.3049	5.9981	MF	Mixed Forests	Tall
BR-CST	-7.9682	-38.3842	DNF	Needleleaf Forests	Tall
BR-Npw	-16.4980	-56.4120	WSA	Shrub and Grasslands	Short

BW-Gum	-18.9647	22.3711	WET	Wetlands	Short
BW-Nxr	-19.5481	23.1792	GRA	Shrub and Grasslands	Short
CA-ARB	52.6950	-83.9452	WET	Wetlands	Short
CA-ARF	52.7008	-83.9550	WET	Wetlands	Short
CA-CF1	58.6658	-93.8300	WET	Wetlands	Short
CA-CF2	58.6658	-93.8300	WET	Wetlands	Short
CA-Ca1	49.8673	-125.3336	ENF	Needleleaf Forests	Tall
CA-Ca2	49.8705	-125.2909	ENF	Needleleaf Forests	Tall
CA-Cbo	44.3167	-79.9333	DBF	Broadleaf Forests	Tall
CA-Cha	45.8847	-67.3569	MF	Mixed Forests	Tall
CA-DBB	49.1293	-122.9849	WET	Wetlands	Short
CA-ER1	43.6405	-80.4123	CRO	Croplands	Short
CA-Gro	48.2167	-82.1556	MF	Mixed Forests	Tall
CA-HPC	68.3203	-133.5188	ENF	Needleleaf Forests	Tall
CA-LP1	55.1119	-122.8414	ENF	Needleleaf Forests	Tall
CA-Let	49.7093	-112.9402	GRA	Shrub and Grasslands	Short
CA-MA1	50.1645	-97.8762	CRO	Croplands	Short
CA-MA2	50.1710	-97.8762	GRA	Shrub and Grasslands	Short
CA-MA3	50.1774	-97.8686	GRA	Shrub and Grasslands	Short
CA-Man	55.8796	-98.4808	ENF	Needleleaf Forests	Tall
CA-NS1	55.8792	-98.4839	ENF	Needleleaf Forests	Tall
CA-NS2	55.9058	-98.5247	ENF	Needleleaf Forests	Tall
CA-NS3	55.9117	-98.3822	ENF	Needleleaf Forests	Tall

CA-NS4	55.9144	-98.3806	ENF	Needleleaf Forests	Tall
CA-NS5	55.8631	-98.4850	ENF	Needleleaf Forests	Tall
CA-NS6	55.9167	-98.9644	OSH	Shrub and Grasslands	Short
CA-NS7	56.6358	-99.9483	OSH	Shrub and Grasslands	Short
CA-NS8	55.8981	-98.2161	ENF	Needleleaf Forests	Tall
CA-Na1	46.4722	-67.1000	ENF	Needleleaf Forests	Tall
CA-Oas	53.6289	-106.1978	DBF	Broadleaf Forests	Tall
CA-Obs	53.9872	-105.1178	ENF	Needleleaf Forests	Tall
CA-Ojp	53.9163	-104.6920	ENF	Needleleaf Forests	Tall
CA-Qcu	49.2671	-74.0365	ENF	Needleleaf Forests	Tall
CA-SCB	61.3089	-121.2984	WET	Wetlands	Short
CA-SCC	61.3079	-121.2992	ENF	Needleleaf Forests	Tall
CA-SF1	54.4850	-105.8176	ENF	Needleleaf Forests	Tall
CA-SF2	54.2539	-105.8775	ENF	Needleleaf Forests	Tall
CA-SJ2	53.9450	-104.6490	ENF	Needleleaf Forests	Tall
CA-SJ3	53.8758	-104.6453	ENF	Needleleaf Forests	Tall
CA-SMC	63.1534	-123.2522	ENF	Needleleaf Forests	Tall
CA-TP2	42.7744	-80.4588	ENF	Needleleaf Forests	Tall
CA-TVC	68.7462	-133.5017	OSH	Shrub and Grasslands	Short
CA-WP1	54.9538	-112.4670	WET	Wetlands	Short
CA-WP2	55.5375	-112.3343	WET	Wetlands	Short
CA-WP3	54.4700	-113.3200	WET	Wetlands	Short
CH-Aws	46.5832	9.7904	GRA	Shrub and Grasslands	Short

CH-Cha	47.2102	8.4104	GRA	Shrub and Grasslands	Short
CH-Dav	46.8153	9.8559	ENF	Needleleaf Forests	Tall
CH-Fru	47.1158	8.5378	GRA	Shrub and Grasslands	Short
CH-Lae	47.4783	8.3644	MF	Mixed Forests	Tall
CH-Oe1	47.2858	7.7319	GRA	Shrub and Grasslands	Short
CH-Oe2	47.2864	7.7338	CRO	Croplands	Short
CN-Cha	42.4025	128.0958	MF	Mixed Forests	Tall
CN-Cng	44.5934	123.5092	GRA	Shrub and Grasslands	Short
CN-Dan	30.4978	91.0664	GRA	Shrub and Grasslands	Short
CN-Din	23.1733	112.5361	EBF	Broadleaf Forests	Tall
CN-Du2	42.0467	116.2836	GRA	Shrub and Grasslands	Short
CN-Ha2	37.6086	101.3269	WET	Wetlands	Short
CN-HaM	37.3700	101.1800	GRA	Shrub and Grasslands	Short
CN-Hgu	32.8453	102.5900	GRA	Shrub and Grasslands	Short
CN-Qia	26.7414	115.0581	ENF	Needleleaf Forests	Tall
CN-Sw2	41.7902	111.8971	GRA	Shrub and Grasslands	Short
CZ-BK1	49.5021	18.5369	ENF	Needleleaf Forests	Tall
CZ-BK2	49.4944	18.5429	GRA	Shrub and Grasslands	Short
CZ-wet	49.0247	14.7704	WET	Wetlands	Short
DE-Akm	53.8662	13.6834	WET	Wetlands	Short
DE-Geb	51.0997	10.9146	CRO	Croplands	Short
DE-Gri	50.9498	13.5126	GRA	Shrub and Grasslands	Short
DE-Hai	51.0792	10.4522	DBF	Broadleaf Forests	Tall

DE-HoH	52.0866	11.2224	DBF	Broadleaf Forests	Tall
DE-Hte	54.2103	12.1761	WET	Wetlands	Short
DE-Kli	50.8931	13.5224	CRO	Croplands	Short
DE-Lkb	49.0996	13.3047	ENF	Needleleaf Forests	Tall
DE-Lnf	51.3282	10.3678	DBF	Broadleaf Forests	Tall
DE-Obe	50.7867	13.7213	ENF	Needleleaf Forests	Tall
DE-RuR	50.6219	6.3041	GRA	Shrub and Grasslands	Short
DE-RuS	50.8659	6.4472	CRO	Croplands	Short
DE-Seh	50.8706	6.4497	CRO	Croplands	Short
DE-SfN	47.8064	11.3275	WET	Wetlands	Short
DE-Spw	51.8923	14.0337	WET	Wetlands	Short
DE-Tha	50.9626	13.5652	ENF	Needleleaf Forests	Tall
DE-Zrk	53.8759	12.8890	WET	Wetlands	Short
DK-Eng	55.6905	12.1918	GRA	Shrub and Grasslands	Short
DK-Sor	55.4859	11.6446	DBF	Broadleaf Forests	Tall
ES-Abr	38.7018	-6.7859	SAV	Shrub and Grasslands	Short
ES-LJu	36.9266	-2.7521	OSH	Shrub and Grasslands	Short
ES-LM1	39.9427	-5.7787	SAV	Shrub and Grasslands	Short
ES-LM2	39.9346	-5.7759	SAV	Shrub and Grasslands	Short
ES-LgS	37.0979	-2.9658	OSH	Shrub and Grasslands	Short
FI-Hyy	61.8474	24.2948	ENF	Needleleaf Forests	Tall
FI-Jok	60.8986	23.5135	CRO	Croplands	Short
FI-Let	60.6418	23.9595	ENF	Needleleaf Forests	Tall

FI-Lom	67.9972	24.2092	WET	Wetlands	Short
FI-Si2	61.8372	24.1967	WET	Wetlands	Short
FI-Sii	61.8327	24.1929	WET	Wetlands	Short
FI-Sod	67.3624	26.6386	ENF	Needleleaf Forests	Tall
FI-Var	67.7549	29.6100	ENF	Needleleaf Forests	Tall
FR-Aur	43.5496	1.1061	CRO	Croplands	Short
FR-Bil	44.4937	-0.9561	ENF	Needleleaf Forests	Tall
FR-EM2	49.8721	3.0207	CRO	Croplands	Short
FR-Fon	48.4764	2.7801	DBF	Broadleaf Forests	Tall
FR-Gri	48.8442	1.9519	CRO	Croplands	Short
FR-Hes	48.6741	7.0647	DBF	Broadleaf Forests	Tall
FR-LBr	44.7171	-0.7693	ENF	Needleleaf Forests	Tall
FR-LGt	47.3229	2.2841	WET	Wetlands	Short
FR-Lgt	47.3229	2.2841	WET	Wetlands	Short
FR-Mej	48.1184	-1.7964	GRA	Shrub and Grasslands	Short
FR-Pue	43.7413	3.5957	EBF	Broadleaf Forests	Tall
GL-NuF	64.1308	-51.3861	WET	Wetlands	Short
ID-Pag	-2.3200	113.9000	EBF	Broadleaf Forests	Tall
IT-CA1	42.3804	12.0266	DBF	Broadleaf Forests	Tall
IT-CA2	42.3772	12.0260	CRO	Croplands	Short
IT-CA3	42.3800	12.0222	DBF	Broadleaf Forests	Tall
IT-Cas	45.0700	8.7175	CRO	Croplands	Short
IT-Col	41.8494	13.5881	DBF	Broadleaf Forests	Tall

IT-Isp	45.8126	8.6336	DBF	Broadleaf Forests	Tall
IT-Lav	45.9562	11.2813	ENF	Needleleaf Forests	Tall
IT-Lsn	45.7405	12.7503	OSH	Shrub and Grasslands	Short
IT-PT1	45.2009	9.0610	DBF	Broadleaf Forests	Tall
IT-Ren	46.5869	11.4337	ENF	Needleleaf Forests	Tall
IT-Ro1	42.4081	11.9300	DBF	Broadleaf Forests	Tall
IT-Ro2	42.3903	11.9209	DBF	Broadleaf Forests	Tall
IT-SR2	43.7320	10.2909	ENF	Needleleaf Forests	Tall
IT-SRo	43.7279	10.2844	ENF	Needleleaf Forests	Tall
IT-Tor	45.8444	7.5781	GRA	Shrub and Grasslands	Short
JP-BBY	43.3230	141.8107	WET	Wetlands	Short
JP-MBF	44.3869	142.3186	DBF	Broadleaf Forests	Tall
JP-Mse	36.0539	140.0269	CRO	Croplands	Short
JP-SMF	35.2617	137.0788	MF	Mixed Forests	Tall
KR-CRK	38.2013	127.2506	CRO	Croplands	Short
MX-Aog	26.9968	-108.7892	DBF	Broadleaf Forests	Tall
MX-EMg	32.0298	-116.6045	CSH	Shrub and Grasslands	Short
MX-Lpa	24.1292	-110.4380	OSH	Shrub and Grasslands	Short
MX-Tes	27.8423	-109.2989	DBF	Broadleaf Forests	Tall
MY-MLM	1.4536	111.1495	EBF	Broadleaf Forests	Tall
MY-PSO	2.9730	102.3062	EBF	Broadleaf Forests	Tall
NL-Hor	52.2404	5.0713	GRA	Shrub and Grasslands	Short
NL-Loo	52.1666	5.7436	ENF	Needleleaf Forests	Tall

NZ-Kop	-37.3879	175.5539	EBF	Broadleaf Forests	Tall
PE-QFR	-3.8344	-73.3190	WET	Wetlands	Short
PH-RiF	14.1412	121.2653	CRO	Croplands	Short
RU-Ch2	68.6169	161.3509	WET	Wetlands	Short
RU-Che	68.6130	161.3414	WET	Wetlands	Short
RU-Cok	70.8291	147.4943	OSH	Shrub and Grasslands	Short
RU-Fy2	56.4476	32.9019	ENF	Needleleaf Forests	Tall
RU-Fyo	56.4615	32.9221	ENF	Needleleaf Forests	Tall
RU-Ha1	54.7252	90.0022	GRA	Shrub and Grasslands	Short
SD-Dem	13.2829	30.4783	SAV	Shrub and Grasslands	Short
SE-Deg	64.1820	19.5565	WET	Wetlands	Short
SE-Htm	56.0976	13.4190	ENF	Needleleaf Forests	Tall
SE-Nor	60.0865	17.4795	ENF	Needleleaf Forests	Tall
SE-Svb	64.2561	19.7745	ENF	Needleleaf Forests	Tall
SJ-Adv	78.1860	15.9230	WET	Wetlands	Short
US-A03	70.4953	-149.8823	BSV	Shrub and Grasslands	Short
US-A32	36.8193	-97.8198	GRA	Shrub and Grasslands	Short
US-A74	36.8085	-97.5489	CRO	Croplands	Short
US-ADR	36.7653	-116.6933	BSV	Shrub and Grasslands	Short
US-ALQ	46.0308	-89.6067	WET	Wetlands	Short
US-AR1	36.4267	-99.4200	GRA	Shrub and Grasslands	Short
US-AR2	36.6358	-99.5975	GRA	Shrub and Grasslands	Short
US-ARb	35.5497	-98.0402	GRA	Shrub and Grasslands	Short

US-ARc	35.5465	-98.0400	GRA	Shrub and Grasslands	Short
US-ASH	36.1697	-120.2010	DBF	Broadleaf Forests	Tall
US-ASL	36.9466	-120.1024	DBF	Broadleaf Forests	Tall
US-ASM	36.1777	-120.2026	DBF	Broadleaf Forests	Tall
US-An1	68.9900	-150.2800	OSH	Shrub and Grasslands	Short
US-An2	68.9500	-150.2100	OSH	Shrub and Grasslands	Short
US-An3	68.9300	-150.2700	OSH	Shrub and Grasslands	Short
US-Atq	70.4696	-157.4089	WET	Wetlands	Short
US-Aud	31.5907	-110.5104	GRA	Shrub and Grasslands	Short
US-BMM	45.7830	-110.7776	GRA	Shrub and Grasslands	Short
US-BZB	64.6955	-148.3208	WET	Wetlands	Short
US-BZF	64.7037	-148.3133	WET	Wetlands	Short
US-BZS	64.6963	-148.3235	ENF	Needleleaf Forests	Tall
US-Bi1	38.0992	-121.4993	CRO	Croplands	Short
US-Bi2	38.1091	-121.5351	CRO	Croplands	Short
US-Bkg	44.3453	-96.8362	GRA	Shrub and Grasslands	Short
US-Blk	44.1580	-103.6500	ENF	Needleleaf Forests	Tall
US-Blo	38.8953	-120.6328	ENF	Needleleaf Forests	Tall
US-Bn1	63.9198	-145.3782	ENF	Needleleaf Forests	Tall
US-Bn2	63.9198	-145.3782	DBF	Broadleaf Forests	Tall
US-Bn3	63.9227	-145.7442	OSH	Shrub and Grasslands	Short
US-Bo1	40.0062	-88.2904	CRO	Croplands	Short
US-Bo2	40.0090	-88.2900	CRO	Croplands	Short

US-Br1	41.9749	-93.6906	CRO	Croplands	Short
US-Br3	41.9747	-93.6936	CRO	Croplands	Short
US-CF1	46.7815	-117.0821	CRO	Croplands	Short
US-CF2	46.7840	-117.0908	CRO	Croplands	Short
US-CF3	46.7551	-117.1261	CRO	Croplands	Short
US-CF4	46.7518	-117.1285	CRO	Croplands	Short
US-CMW	31.6637	-110.1777	DBF	Broadleaf Forests	Tall
US-CPk	41.0680	-106.1187	ENF	Needleleaf Forests	Tall
US-CRT	41.6285	-83.3471	CRO	Croplands	Short
US-CZ2	37.0311	-119.2566	ENF	Needleleaf Forests	Tall
US-CZ3	37.0674	-119.1951	ENF	Needleleaf Forests	Tall
US-CZ4	37.0675	-118.9867	ENF	Needleleaf Forests	Tall
US-CaV	39.0633	-79.4208	GRA	Shrub and Grasslands	Short
US-Ced	39.8379	-74.3791	CSH	Shrub and Grasslands	Short
US-ChR	35.9311	-84.3324	DBF	Broadleaf Forests	Tall
US-Cop	38.0900	-109.3900	GRA	Shrub and Grasslands	Short
US-Ctn	43.9500	-101.8466	GRA	Shrub and Grasslands	Short
US-Cwt	35.0592	-83.4275	DBF	Broadleaf Forests	Tall
US-DFC	43.3448	-89.7117	CRO	Croplands	Short
US-DPW	28.0521	-81.4361	WET	Wetlands	Short
US-Dia	37.6773	-121.5296	GRA	Shrub and Grasslands	Short
US-Dix	39.9712	-74.4346	MF	Mixed Forests	Tall
US-Dk1	35.9712	-79.0934	GRA	Shrub and Grasslands	Short

US-Dk2	35.9736	-79.1004	DBF	Broadleaf Forests	Tall
US-Dk3	35.9782	-79.0942	ENF	Needleleaf Forests	Tall
US-EML	63.8784	-149.2536	OSH	Shrub and Grasslands	Short
US-Elm	25.5519	-80.7826	WET	Wetlands	Short
US-Esm	25.4379	-80.5946	WET	Wetlands	Short
US-FPe	48.3077	-105.1019	GRA	Shrub and Grasslands	Short
US-FR2	29.9495	-97.9962	WSA	Shrub and Grasslands	Short
US-FR3	29.9400	-97.9900	CSH	Shrub and Grasslands	Short
US-Fcr	65.3968	-148.9348	OSH	Shrub and Grasslands	Short
US-Fmf	35.1426	-111.7273	ENF	Needleleaf Forests	Tall
US-Fuf	35.0890	-111.7620	ENF	Needleleaf Forests	Tall
US-Fwf	35.4454	-111.7718	GRA	Shrub and Grasslands	Short
US-GBT	41.3658	-106.2397	ENF	Needleleaf Forests	Tall
US-GMF	41.9667	-73.2333	MF	Mixed Forests	Tall
US-Goo	34.2547	-89.8735	GRA	Shrub and Grasslands	Short
US-HBK	43.9397	-71.7181	DBF	Broadleaf Forests	Tall
US-HRA	34.5852	-91.7517	CRO	Croplands	Short
US-HRC	34.5888	-91.7517	CRO	Croplands	Short
US-Ha1	42.5378	-72.1715	DBF	Broadleaf Forests	Tall
US-Hn2	46.6889	-119.4641	GRA	Shrub and Grasslands	Short
US-Hn3	46.6878	-119.4614	OSH	Shrub and Grasslands	Short
US-Ho2	45.2091	-68.7470	ENF	Needleleaf Forests	Tall
US-Ho3	45.2072	-68.7250	ENF	Needleleaf Forests	Tall

US-IB1	41.8593	-88.2227	CRO	Croplands	Short
US-IB2	41.8406	-88.2410	GRA	Shrub and Grasslands	Short
US-ICh	68.6068	-149.2958	OSH	Shrub and Grasslands	Short
US-ICs	68.6058	-149.3110	WET	Wetlands	Short
US-ICt	68.6063	-149.3041	OSH	Shrub and Grasslands	Short
US-Ivo	68.4865	-155.7503	WET	Wetlands	Short
US-Jo1	32.5820	-106.6350	OSH	Shrub and Grasslands	Short
US-Jo2	32.5849	-106.6032	OSH	Shrub and Grasslands	Short
US-KL1	42.4847	-85.4422	CRO	Croplands	Short
US-KL2	42.4767	-85.4467	CRO	Croplands	Short
US-KL3	42.4736	-85.4474	CRO	Croplands	Short
US-KM1	42.4376	-85.3287	GRA	Shrub and Grasslands	Short
US-KM2	42.4427	-85.3104	CRO	Croplands	Short
US-KM3	42.4440	-85.3097	GRA	Shrub and Grasslands	Short
US-KM4	42.4423	-85.3301	CRO	Croplands	Short
US-KUT	44.9950	-93.1863	GRA	Shrub and Grasslands	Short
US-LA1	29.5013	-90.4449	WET	Wetlands	Short
US-LA2	29.8587	-90.2869	WET	Wetlands	Short
US-LL1	31.2792	-84.5329	SAV	Shrub and Grasslands	Short
US-LL2	31.2010	-84.4449	SAV	Shrub and Grasslands	Short
US-LL3	31.2688	-84.4787	SAV	Shrub and Grasslands	Short
US-LPH	42.5419	-72.1850	DBF	Broadleaf Forests	Tall
US-LS1	31.5615	-110.1403	GRA	Shrub and Grasslands	Short

US-LS2	31.5659	-110.1344	SAV	Shrub and Grasslands	Short
US-Lin	36.3566	-119.8423	CRO	Croplands	Short
US-MBP	47.5051	-93.4893	WET	Wetlands	Short
US-MC1	48.1873	-114.1548	CRO	Croplands	Short
US-MH1	45.9206	-108.2414	CRO	Croplands	Short
US-MRM	40.8164	-74.0435	WET	Wetlands	Short
US-MRf	44.6465	-123.5515	ENF	Needleleaf Forests	Tall
US-MSR	47.4758	-111.7207	CRO	Croplands	Short
US-MWA	42.2143	-84.8539	CRO	Croplands	Short
US-MWF	42.3660	-85.3526	MF	Mixed Forests	Tall
US-Me2	44.4523	-121.5574	ENF	Needleleaf Forests	Tall
US-Me3	44.3154	-121.6078	ENF	Needleleaf Forests	Tall
US-Me6	44.3233	-121.6078	ENF	Needleleaf Forests	Tall
US-Mj1	46.9948	-109.6137	CRO	Croplands	Short
US-Mj2	46.9957	-109.6295	CRO	Croplands	Short
US-Mpj	34.4385	-106.2377	WSA	Shrub and Grasslands	Short
US-MtB	32.4164	-110.7256	ENF	Needleleaf Forests	Tall
US-Myb	37.0500	-121.7651	WET	Wetlands	Short
US-NGC	64.8614	-163.7008	GRA	Shrub and Grasslands	Short
US-ONA	27.3836	-81.9509	GRA	Shrub and Grasslands	Short
US-ORv	40.0201	-83.0183	WET	Wetlands	Short
US-OWC	41.3795	-82.5125	WET	Wetlands	Short
US-Oho	41.5545	-83.8438	DBF	Broadleaf Forests	Tall

US-PFa	45.9459	-90.2723	MF	Mixed Forests	Tall
US-PHM	42.7423	-70.8301	WET	Wetlands	Short
US-PSH	36.2347	-119.9247	DBF	Broadleaf Forests	Tall
US-PSL	36.8276	-120.1397	DBF	Broadleaf Forests	Tall
US-Rls	43.1439	-116.7356	CSH	Shrub and Grasslands	Short
US-Rms	43.0645	-116.7486	CSH	Shrub and Grasslands	Short
US-Ro1	44.7143	-93.0898	CRO	Croplands	Short
US-Ro2	44.7288	-93.0888	CRO	Croplands	Short
US-Ro3	44.7217	-93.0893	CRO	Croplands	Short
US-Ro4	44.6781	-93.0723	GRA	Shrub and Grasslands	Short
US-Ro5	44.6910	-93.0576	CRO	Croplands	Short
US-Ro6	44.6946	-93.0578	CRO	Croplands	Short
US-Rpf	65.1198	-147.4290	DBF	Broadleaf Forests	Tall
US-Rwe	43.0653	-116.7591	CSH	Shrub and Grasslands	Short
US-Rwf	43.1207	-116.7231	CSH	Shrub and Grasslands	Short
US-Rws	43.1675	-116.7132	OSH	Shrub and Grasslands	Short
US-SCd	33.6518	-116.3721	BSV	Shrub and Grasslands	Short
US-SCf	33.8079	-116.7717	MF	Mixed Forests	Tall
US-SCg	33.7365	-117.6946	GRA	Shrub and Grasslands	Short
US-SCs	33.7343	-117.6959	OSH	Shrub and Grasslands	Short
US-SCw	33.6047	-116.4527	OSH	Shrub and Grasslands	Short
US-SFP	43.2408	-96.9020	CRO	Croplands	Short
US-SO2	33.3738	-116.6228	CSH	Shrub and Grasslands	Short

US-SO3	33.3771	-116.6226	CSH	Shrub and Grasslands	Short
US-SO4	33.3845	-116.6406	CSH	Shrub and Grasslands	Short
US-SP1	29.7381	-82.2188	ENF	Needleleaf Forests	Tall
US-SP2	29.7648	-82.2448	ENF	Needleleaf Forests	Tall
US-SP3	29.7548	-82.1633	ENF	Needleleaf Forests	Tall
US-SRC	31.9083	-110.8395	OSH	Shrub and Grasslands	Short
US-SRG	31.7894	-110.8277	GRA	Shrub and Grasslands	Short
US-SRM	31.8214	-110.8661	WSA	Shrub and Grasslands	Short
US-SRS	31.8173	-110.8508	WSA	Shrub and Grasslands	Short
US-SdH	42.0693	-101.4072	GRA	Shrub and Grasslands	Short
US-Seg	34.3623	-106.7020	GRA	Shrub and Grasslands	Short
US-Ses	34.3349	-106.7442	OSH	Shrub and Grasslands	Short
US-Skr	25.3629	-81.0776	EBF	Broadleaf Forests	Tall
US-Slt	39.9138	-74.5960	DBF	Broadleaf Forests	Tall
US-Snd	38.0366	-121.7540	GRA	Shrub and Grasslands	Short
US-Sne	38.0369	-121.7547	GRA	Shrub and Grasslands	Short
US-Snf	38.0402	-121.7272	GRA	Shrub and Grasslands	Short
US-Srr	38.2006	-122.0264	WET	Wetlands	Short
US-Sta	41.3966	-106.8024	OSH	Shrub and Grasslands	Short
US-Tw1	38.1074	-121.6469	WET	Wetlands	Short
US-Tw3	38.1159	-121.6467	CRO	Croplands	Short
US-Tw4	38.1030	-121.6414	WET	Wetlands	Short
US-Tw5	38.1072	-121.6426	WET	Wetlands	Short

US-Twt	38.1077	-121.6528	CRO	Croplands	Short
US-UMB	45.5598	-84.7138	DBF	Broadleaf Forests	Tall
US-UMd	45.5625	-84.6975	DBF	Broadleaf Forests	Tall
US-Uaf	64.8663	-147.8555	ENF	Needleleaf Forests	Tall
US-Var	38.4133	-120.9507	GRA	Shrub and Grasslands	Short
US-WCr	45.8059	-90.0799	DBF	Broadleaf Forests	Tall
US-WPT	41.4646	-82.9962	WET	Wetlands	Short
US-Whs	31.7438	-110.0522	OSH	Shrub and Grasslands	Short
US-Wi2	46.6869	-91.1528	ENF	Needleleaf Forests	Tall
US-Wi3	46.6347	-91.0987	DBF	Broadleaf Forests	Tall
US-Wi4	46.7393	-91.1663	ENF	Needleleaf Forests	Tall
US-Wi5	46.6531	-91.0858	ENF	Needleleaf Forests	Tall
US-Wi7	46.6491	-91.0693	OSH	Shrub and Grasslands	Short
US-Wi9	46.6188	-91.0814	ENF	Needleleaf Forests	Tall
US-Wkg	31.7365	-109.9419	GRA	Shrub and Grasslands	Short

Supplementary Table 4: Meta data of the 90 sap flow measurement stations from SAPFLUXNET database used in this study for training and validation of the deep learning models of the tall vegetation transpiration stress factor

**Site ID**	**Latitude**	**Longitude**
ARG_MAZ	-51,5794	-72,2864
ARG_TRE	-51,3167	-72,1850
AUS_CAN_ST1_EUC	-37,5800	149,1700

AUS_CAR_THI_00F	-38,3830	146,6830
AUS_ELL_HB_HIG	-36,7850	146,5820
AUS_MAR_HSD_HIG	-37,6430	145,5810
AUS_MAR_UBW	-37,8880	145,5720
AUS_RIC_EUC_ELE	-33,6178	150,7403
AUS_WOM	-37,4222	144,0944
AUT_TSC	47,2333	10,8389
BRA_CAX_CON	-1,7923	-51,4340
CAN_TUR_P39_POS	42,7098	-80,3574
CHE_DAV_SEE	46,8167	9,8562
CHE_LOT_NOR	46,3918	7,7613
CHE_PFY_CON	46,3000	7,6000
CHN_ARG_GWD	40,7542	89,9856
CHN_ARG_GWS	41,3817	89,9383
CHN_HOR_AFF	42,7167	122,3667
CHN_YIN_ST1	42,4544	85,7167
CHN_YIN_ST2_DRO	42,1144	85,1311
CHN_YIN_ST3_DRO	42,2883	85,9883
COL_MAC_SAF_RAD	1,5011	-75,3611
CRI_TAM_TOW	10,3869	-84,6258
CZE_BIK	49,4944	18,5300
CZE_BIL_BIL	49,2485	16,6869
CZE_KRT_KRT	49,3223	16,7486

CZE_LAN	48,6817	16,9464
CZE_LIZ_LES	49,0667	13,6797
CZE_STI	49,0358	17,9700
DEU_MER_BEE_NON	49,2739	7,8074
DEU_STE_2P3	53,1000	13,0000
ESP_ALT_ARM	40,7769	-2,3283
ESP_ALT_TRI	40,8044	-2,2328
ESP_CAN	41,4310	2,0736
ESP_GUA_VAL	40,8953	-4,0339
ESP_LAH_COM	37,7400	-3,3800
ESP_LAS	28,3060	-16,5683
ESP_MAJ_MAI	39,9415	-5,7734
ESP_MON_SIE_NAT	41,1167	-3,5000
ESP_RIN	40,6003	-6,0167
ESP_RON_PIL	36,6929	-5,0196
ESP_SAN_A2_45I	37,2491	-5,7971
ESP_TIL_MIX	41,3326	1,0144
ESP_VAL_BAR	42,2029	1,8205
ESP_YUN_C1	36,7246	-4,9689
FIN_HYY_SME	61,8474	24,2948
FIN_PET	69,4918	27,2311
FRA_FON	48,4763	2,7801
FRA_HES_HE2_NON	48,6742	7,0647

FRA_PUE	43,7414	3,5958
GBR_GUI_ST1	57,2667	-4,8167
GUF_GUY_GUY	5,2788	-52,9249
HUN_SIK	47,9273	20,4444
IDN_JAM_OIL	-2,0709	102,7918
IDN_PON_STE	-1,4940	120,0570
ISR_YAT_YAT	31,3450	35,0515
ITA_TOR	45,8238	7,5609
JPN_EBE_HYB	43,0761	141,5230
KOR_TAE_TC1_LOW	37,3048	127,3175
MDG_SEM_TAL	-18,9317	48,7117
MDG_YOU_SHO	-18,9472	48,3953
MEX_COR_YP	19,4931	-97,0422
NLD_LOO	52,1665	5,7436
NLD_SPE_DOU	52,2511	5,6903
NZL_HUA_HUA	-36,7958	174,4903
PRT_LEZ_ARN	38,8333	-8,8167
RUS_FYO	56,4615	32,9221
SWE_NOR_ST1_AF1	60,0860	17,4800
SWE_SKO_MIN	58,3639	12,1498
SWE_SVA_MIX_NON	64,2561	19,7745
THA_KHU	15,2730	103,0810
USA_CHE_ASP	45,9370	-90,2670

USA_DUK_HAR	36,9782	-79,0942
USA_HIL_HF1_POS	36,2173	-78,8642
USA_HUY_LIN_NON	42,5275	-74,1572
USA_MOR_SF	39,3230	-86,4133
USA_NWH	34,5789	-91,2564
USA_ORN_ST1_AMB	35,9000	-84,3330
USA_PAR_FER	35,8031	-76,6679
USA_PER_PER	30,2061	-83,8700
USA_PJS_P04_AMB	34,3864	-106,5294
USA_SIL_OAK_1PR	39,9156	-74,5956
USA_SMI_SCB	38,8935	-78,1454
USA_SMI_SER	38,8890	-76,5590
USA_SWH	34,1089	-91,1253
USA_SYL_HL1	46,2420	-89,3477
USA_UMB_CON	45,5597	-84,7133
USA_WIL_WC2	45,8131	-90,0867
ZAF_NOO_E3_IRR	-33,2010	19,3375
ZAF_WEL_SOR	-33,4817	18,9560

References

1. Wei, Z. *et al.* Revisiting the contribution of transpiration to global terrestrial evapotranspiration. *Geophysical Research Letters* **44**, 2792–2801 (2017). URL `https://agupubs.`

onlinelibrary.wiley.com/doi/abs/10.1002/2016GL072235.

2. Wielicki, B. A. *et al.* Clouds and the earth's radiant energy system (ceres): An earth observing system experiment. *Bulletin of the American Meteorological Society* **77**, 853–868 (1996).

3. Aumann, H. *et al.* Airs/amsu/hsb on the aqua mission: design, science objectives, data products, and processing systems. *IEEE Transactions on Geoscience and Remote Sensing* **41**, 253–264 (2003).

4. Beck, H. E. *et al.* Mswep: 3-hourly 0.25 global gridded precipitation (1979–2015) by merging gauge, satellite, and reanalysis data. *Hydrology and Earth System Sciences* **21**, 589–615 (2017). URL https://hess.copernicus.org/articles/21/589/2017/.

5. Pulliainen, J. *et al.* Patterns and trends of northern hemisphere snow mass from 1980 to 2018. *Nature* **581**, 294–298 (2020). URL https://doi.org/10.1038/s41586-020-2258-0.

6. Moesinger, L. *et al.* The global long-term microwave vegetation optical depth climate archive (vodca). *Earth System Science Data* **12**, 177–196 (2020). URL https://essd.copernicus.org/articles/12/177/2020/.

7. Dorigo, W. *et al.* Esa cci soil moisture for improved earth system understanding: State-of-the art and future directions. *Remote Sensing of Environment* **203**, 185–215 (2017). URL https://www.sciencedirect.com/science/article/pii/S0034425717303061. Earth Observation of Essential Climate Variables.

8. Ramachandran, P., Zoph, B. & Le, Q. V. Searching for activation functions (2017). arXiv:1710.05941.

9. Hendrycks, D. & Gimpel, K. Gaussian error linear units (gelus) (2020). arXiv:1606.08415.
